# Medicinal Plants in Food Supplements for Gastrointestinal Disorders: Critical Assessment of Health Claims on Gastric Acid Regulation

**DOI:** 10.3390/nu17233674

**Published:** 2025-11-24

**Authors:** Renāte Teterovska, Rūta Elvīra Skotele, Baiba Maurina, Inga Sile

**Affiliations:** 1 Department of Pharmacology and Pharmacotherapy, Riga Stradinš University, 16 Dzirciema Street, LV-1007 Riga, Latvia; 044180@rsu.edu.lv; 2 Baltic Biomaterials Centre of Excellence, Headquarters at Riga Technical University, LV-1658 Riga, Latvia; 3 Department of Applied Pharmacy, Riga Stradinš University, 16 Dzirciema Street, LV-1007 Riga, Latvia; baiba.maurina@rsu.lv (B.M.); inga.sile@rsu.lv (I.S.)

**Keywords:** medicinal plants, gastrointestinal disorders, gastric acid, food supplements, health claims

## Abstract

Background: Gastrointestinal (GI) disorders associated with increased gastric acid secretion, such as gastroesophageal reflux, dyspepsia, bloating, and abdominal pain, significantly impair quality of life and present a substantial healthcare burden. Conventional therapies may have limited efficacy or undesirable side effects, underscoring the need for safe complementary approaches. This study systematically identifies and reviews the medicinal plants used in food supplements (FSs) marketed in Latvia for digestive health, focusing on the conditions linked to excess gastric acid. Methods: A structured literature search was conducted to identify European plant species with proven protective effects on the digestive system or the ability to influence gastric acid levels. A market analysis was performed using the Latvian Food and Veterinary Service FS Register. Results: A total of 218 FS-containing medicinal plants were identified, of which 15 species were included in at least ten products. The most frequently used plants were peppermint (*Mentha piperita*), artichoke (*Cynara cardunculus*), fennel (*Foeniculum vulgare*), Milk thistle (*Silybum marianum*), dandelion (*Taraxacum officinale*), chamomile (*Matricaria chamomilla*), psyllium (*Plantago ovata*), licorice (*Glycyrrhiza glabra*), caraway (*Carum carvi*), lemon balm (*Melissa officinalis*), and chicory (*Cichorium intybus*). Label claims most often referred to supporting digestion, relieving bloating, and maintaining normal GI function. However, the majority of claims lacked robust clinical substantiation, and were based primarily on traditional use. Discrepancies between product information and available scientific evidence highlight regulatory and consumer protection challenges. Conclusions: This work contributes to the critical evaluation of plant-based FSs for digestive health, emphasizing the need for standardized preparations, harmonized health claim assessment, and further clinical research to establish efficacy and safety.

## 1. Introduction

Health conditions associated with stomach acid include gastroesophageal reflux disease (GERD), esophagitis, dyspepsia, chronic gastritis, gastric and duodenal ulcers, and *Helicobacter pylori* infection. Indigestion or dyspepsia can cause symptoms such as heaviness in the stomach after eating, early satiety and pain or burning in the epigastria, bloating, and nausea. These symptoms make a lower quality of life for patients, so it is important to eliminate them [1,2]. The prevalence of diseases of the digestive tract is high all over the world. More than 40% of people worldwide have functional gastrointestinal (GI) tract disorders. These diseases cause significant suffering, they also affect the quality of life and productivity of patients, and can also be fatal [3,4]. It also represents a significant burden and expenditure on healthcare, as conventional medicine can have no significant therapeutic effect or cause adverse effects [2]. This emphasizes the need for alternative and complementary treatment approaches that can provide lasting relief with fewer adverse effects.

A potential alternative approach is the use of herbal medicinal products (HMPs) and food supplements (FSs) for the prevention and treatment of diseases and symptoms associated with increased stomach acid. Recent research on the treatment of GI tract disorders focuses on the possible role of plant and natural compounds due to their availability, better protection, lower cost, and lower toxicity, so it is important to conduct literature reviews that summarize the properties of plants. Literature reviews have been conducted on plants found in Asia and their effects on stomach acid, but this type of research has not been conducted on medicinal plants found in Europe [5,6]. The use of herbal medicines in the world as a component of complementary and alternative medicine is growing. People use herbal medicines to maintain or improve their health and to improve and treat chronic or acute diseases. Patients choose this method of treatment because they are dissatisfied with the results of traditional medicine treatment and its expensive cost, or because of positive experiences with the use of medicinal plants in the past [7,8].

FSs are used by consumers of all ages and are also intended for both healthy people and people with health problems. The most of popular FSs contain herbal ingredients: medicinal plants and their extracts [9,10]. Medicinal plants are rich in active metabolites in roots, bark, leaves, flowers, fruits, and seeds and have been used for medicinal purposes since ancient times. Over-the-counter and prescription medicines of plant origin are strictly regulated and use purified ingredients, whereas the production of herbal medicinal products (HMPs) and food supplements is less regulated [9,11]. This means that medicinal plants or parts of plants used in FSs may vary in consistency and concentration, may have poor chemical and microbiological quality, and may be dangerous to patients [10]. Although the European Council has tried to harmonize the market, information and labeling issues linger. The line between herbal medicine and FSs is unclear to consumers and can lead to unapproved or inappropriate use [9,12]. Bilia and Costa [12] have described this issue in detail: in short, HMP safety and efficacy analysis is the company’s responsibility and based on “well-established use”. Traditional herbal medicinal products do not require clinical tests as long as they meet sufficient safety data. FSs are regulated by Directive 2002/46/EC, but very few herbal origin products or active ingredients have been included in the registry of approved health claims for labeling [9,12]. EFSA (European Food Safety Authority) has compiled data on some plant products’ safety, such as adverse effects and genotoxicity, in their Botanical Summary report (available here: https://www.efsa.europa.eu/en/microstrategy/botanical-summary-report accessed on 6 June 2025). However, unlike nutritional components, such as vitamins and minerals, that have a health claim registry, the list of herbal heath claims is still under development [13], and these claims, which are used by companies, go unchecked. Thus, labeling can be misleading to consumers, as it might lack sufficient evidence for the product’s effects or safety information [14].

We aimed to compile FS-containing herbs with proposed effects on GI issues caused by increased stomach acid and analyze labeling information quality and health claims sold in Latvian market by comparing the provided data to normative documents and studies. Therefore, two main goals were set out: Firstly, a review of plants typically used in Europe for GI tract problems was performed to establish a base for market analysis and obtain data on the level of evidence for the most common plants used in FSs. Secondly, a Latvian FS market analysis was executed to compile the health claims on labels, and their quality was evaluated.

## 2. Methods

### 2.1. Literature Selection

A literature review was conducted to identify studies on the effects of medicinal plants on gastrointestinal disorders related to increased gastric acid secretion. Publications were searched using the following keywords: “herbal medicine” OR “plant medicine” OR “medicinal plant” OR “extract” AND (“gastric acid” OR “stomach acid” OR “gastrointestinal diseases” OR “dyspepsia” OR “acid reflux” OR “GERD” OR “indigestion”). Additionally, searches were performed using the names of plants traditionally used in Latvia for gastrointestinal (GI) issues [15], combined with the same keywords. For example, (“Mentha piperita” OR peppermint) AND (“gastric acid” OR “stomach acid” OR “gastrointestinal diseases” OR “dyspepsia” OR “acid reflux” OR “GERD” OR “indigestion”). The exclusion criteria were studies with titles, abstracts, or results that were not relevant to the aim of the research, as well as studies that did not describe the impact of plants on digestive system disorders related to increased gastric acid secretion, including symptoms such as a feeling of heaviness in the stomach after eating, early satiety, pain or burning in the epigastrium, abdominal bloating, and nausea. The species that are not typical of European flora or traditional folk use were excluded from further analysis, as we wanted to focus on the flora of the specific area and on previous reports regarding these plants [16,17,18]. Some effects of popular plants have already been reported in these reviews [17,19]. Although these plants (*Zingiber officinale* Roscoe, *Piper nigrum* L., *Curcuma longa* L., *Emblica officinalis* Gaertn., and various *Aloe* species) are widely used in FSs, our focus was on typical and traditionally used European species. This limits our study to some extent, but provides valuable information on local plants. Studies on the properties of medicinal plants were obtained from the literature in databases such as PubMed and ScienceDirect. Each study was evaluated according to the following criteria: level of evidence (in vitro, in vivo, model, trial, clinical trial, participants), plant preparation (type mentioned), dosage used, posology, and observed effects. Only studies with a clear and detailed methodology were included. The search was conducted independently by two researchers, and the findings were combined.

### 2.2. Market Screening and Label and Health Claim Analysis

To evaluate the availability and characteristics of herbal food supplements targeting digestive disorders in Latvia, a market analysis was conducted. The aim was to identify products containing medicinal plants with potential effects on gastrointestinal conditions related to increased gastric acid secretion. Data were obtained from the Latvian Food and Veterinary Service (Pārtikas un veterinārais dienests, PVD) Food Supplements Register, available via the PVD e-services website: https://pakalpojumi.pvd.gov.lv/lv/supplements accessed on 31 December 2024. Searches were conducted using the Latvian keywords “kuņģis,” “gremošana,” “meteorisms,” “uzpūšanās,” “dedzināšana,” “atvilnis,” “slikta dūša,” “nelabums,” “sāpes vēderā,” and “spazmas,” corresponding, respectively, to the English terms “stomach,” “digestion,” “meteorism,” “bloating,” “burning sensation,” “reflux,” “nausea,” “discomfort,” “abdominal pain,” and “spasms.” To account for Latvian declensions, searches were also performed using truncated keywords with a wildcard (*) to capture different word endings.

Products registered up to 31 December 2024 and containing medicinal plants from the European flora were selected and compiled. These products were included if their labeling indicated effects on digestive system disorders associated with increased stomach acid secretion, such as heaviness in the stomach after eating, early satiety, pain or burning in the epigastrium, bloating, and nausea. More detailed information on all products, the medicinal plants they contain, and the exact wording of their labeling can be accessed in the Dataverse dataset [20].

For the quantitative analysis, only medicinal herbs present in at least 10 products were included. These plants were summarized in a table, indicating their Latvian, English, and Latin names; botanical family; effects on the digestive system; and the number of food supplements in which each medicinal plant was included (Table 1).

## 3. Results

### 3.1. Plants Traditionally Used in Europe to Eliminate Symptoms of Increased Gastric Acid Secretion

Although there are several reviews on gastroprotective plants, few focus specifically on species native to Europe [16,17,18]. However, knowledge about medicinal plants in Central Europe and the Mediterranean has been documented in written sources from ancient times to the present day. Many of the most popular plant species native to Europe are well known in folk medicine for their beneficial effects on digestive system disorders. The dominant medicinal plant families in Europe include the mint family (*Lamiaceae*), the aster family (*Asteraceae*), and the rose family (*Rosaceae*), particularly in Eastern and Central Europe. Among the genera, the most common are peppermint (*Mentha*), oregano (*Origanum*), and thyme (*Thymus*). The most popular of these species include common oregano (*Origanum vulgare* L.); Roman chamomile (*Chamaemelum nobile* (L.) All.) in southern Europe; forest raspberry (*Rubus idaeus* L.) and wild thyme (*Thymus serpyllum* L.) in Eastern Europe; and St. John’s wort (*Hypericum perforatum* L.) in Central Europe [18,21,22,23]. Scientific evidence regarding medicinal plants that affect the digestive tract is presented for the species listed in Table 2.

Latvian folk medicine describes more than 120 medicinal plants used for disorders of the digestive system. For upset stomach and digestive disturbances, the following plants are mentioned: sweet flag (*Acorus calamus* L.), common beet (*Beta vulgaris* L.), wormwood (*Artemisia absinthium* L.), rough horsetail (*Equisetum hyemale* L.), bilberry (*Vaccinium myrtillus* L.), and broad-leaved thyme (*Thymus pulegioides* L.). To relieve burning sensations, plants such as caraway (*Carum carvi* L.) and wormwood have traditionally been used. Yarrow (*Achillea millefolium* L.) and alder buckthorn (*Frangula alnus* Mill.) are mentioned for bloating or meteorism, while chicory (*Cichorium intybus* L.) is used in cases of nausea [15].

These plants are commonly prescribed in cases of generalized abdominal pain and used for sharp throbbing abdominal pain (see Table 3). These are plants of the aster family, such as yarrow, elecampane (*Inula helenium* L.), tansy (*Tanacetum vulgare* L.), southern wormwood (*Artemisia abrotanum* L.), wormwood, mugwort (*Artemisia vulgaris* L.), coltsfoot (*Tussilago farfara* L.), spear thistle (*Cirsium vulgare* (Savi) Ten), and cornflower (*Cyanus segeum* Hill.). Folk medicine references many plants from the mint family, including lemon balm (*Melissa officinalis* L.), peppermint (*Mentha × piperita* L.), broad-leaved thyme, and others [15].

### 3.2. Market Screening and Label Assessment

A search using several different keywords was conducted to retrieve FSs from the registry. The use of the keywords “digestion” and “stomach” for filtering the data retrieved the highest number of products, 591 and 400, respectively. The keywords “meteorism”, “bloating”, “spasms”, and “burning” were also used for data filtering. All registered information was compiled and analyzed. A market review of FSs included in the PVD FS register identified 218 products containing medicinal herbs specific to Europe, whose labeling indicates effects on digestive disorders associated with increased stomach acid. The most common indications were general and did not specify a particular activity of the plant. Typical label information included phrases such as “Supports digestion”, “Contributes to the normal function of intestinal tract”, “Stimulates the digestion”, and “Helps to reduce discomfort”.

From the initial list of FS products, 1024 supplements were excluded. Difficulties in selecting FSs and medicinal plants resulted from the structure of the FS register, which does not allow for searches based on multiple criteria, including indications, contraindications, or adverse effects. Some excluded entries contained only general statements such as “does not affect the digestive system,” “does not irritate the stomach,” or “not to be used in case of gastrointestinal disorders”.

In the European Union, the use of health claims and indications on FS labels is strictly regulated. Health claims are statements that suggest a relationship between food, nutrient, or ingredient and health benefits, and their use is not mandatory. In fact, such claims may be used only if they are registered on official lists or supported by scientific evidence. As the list of herbal health claims has not been fully reviewed or harmonized at the EU level, responsibility for ensuring the accuracy of claims lies with manufacturers. In many cases, indications for use are not provided on product labels, and not all medicinal plants listed in the composition exert the effects indicated. These FSs and medicinal plants were therefore excluded from the market review, which may mean that some products and plants with potential effects on stomach acid and the prevention of related health problems were not considered in this study.

The products featured 307 variations in health claims on their labels. The most frequent claim was “supports digestion”, appearing 24 times. This claim appeared in numerous variations (32), such as “stimulates digestion,” “stimulates normal digestion,” “stimulates the digestion process,” or “supports the function of the digestive system.” Other common claims included phrases like “helps maintain” (12) or “maintains” (11) normal digestion and/or GI function, and related synonyms, altogether accounting for 43 mentions in various forms. There were also numerous variations in health claims referring to promoting or improving digestion or GI function, with 132 and 22 mentions, respectively. Variations included claims about stimulating the secretion of digestive juices, stomach acid, or bile acids, as well as maintaining pH balance. A commonly used health claim related to a positive impact on GI functions and the secretion of various acids, appearing more than 20 times. Other popular health claims concerned bloating, meteorism, reduction in gas, and feelings of heaviness in the stomach. Many product labels also included health claims related to liver function and detoxification. In some cases, specific plants, such as fennel, cinnamon, and liquorice, were explicitly associated with particular health claims.

The products contained more than 100 different plants in FSs. Plants found in more than ten FSs were analyzed in detail and compared with scientific studies. Although many products contained *Zingiber officinale* Roscoe, *Piper nigrum* L., *Curcuma longa* L., *Emblica officinalis* Gaertn., and various *Aloe*, these species are not typical of European flora or traditional folk use and were therefore excluded from further analysis. Their effects are reported in this review [19].

The most common type of preparation was extract (unspecified) followed by powders (unspecified), dry extract, or dried plant material (see Table 4). In most cases, information on the product label was limited and did not reveal the types of extracts or preparation specification, such as extraction solvent, concentration, or end-product (dry, soft or liquid extract). Many product labels did not contain information on plant preparation, but only mentioned the name and plant part in the capsule, tablet, or other drug formulation, thus rendering it impossible to determine the type of plant preparation. This shows another issue: since the registry holder does not need to show precise information on this topic, they do not provide it upon registration. Some contents were provided as the total sum per dosage. Therefore, it was impossible to compare the product to scientific data in full, such as the extract type of effects on biological activity or dosage. Thus, limited analysis could be carried out in this study. In addition, the different plant parts used in products or no data on the plant part on the label led to difficulties in comparing the effects and health claims.

### 3.3. Plant and Health Claim Analysis

#### 3.3.1. Peppermint (*Mentha × piperita* L.)

*Mentha piperita* was the most frequently included plant (35 products) in FSs intended for gastrointestinal problems. The labeling texts of these FSs indicate properties such as having a beneficial effect on the functioning of the digestive system, contributing to normal GI function, improving stomach function, stimulating digestion, and preventing flatulence and abdominal cramps [20]. The EMA states that peppermint preparations can be used for the relief of digestion-related problems, such as indigestion and flatulence. Their indication and safety are based on long-standing traditional use, as there is insufficient evidence from clinical trials [24]. Peppermint belongs to the mint family and is traditionally used for generalized abdominal pain, sharp pulsating pain in the GI tract, and as an appetite stimulant [15,25]. In one clinical trial, 40 drops of peppermint extract were found to reduce gastric residual volume in patients undergoing mechanical ventilation and nasogastric tube feeding in the intensive care unit [25]. However, the highly specific design of this study makes it difficult to extrapolate the findings to the general population.

The essential oil (EO) extracted from peppermint is used in many products. It primarily contains menthol, carvone, other volatile compounds, and polyphenols [26,27,28]. In a clinical trial investigating peppermint oil in children with functional abdominal pain, the effect of EO was dose-dependent, and menthol exposure was shown to affect GI contractility [27]. Menthol activates cold-menthol receptor 1 (TRPM8 channel), which reduces pain in the GI tract, and it also blocks L-type Ca^2+^ channels, producing an antispasmodic effect [21,26,27,28]. Carvone and menthol have a strong bactericidal effect against *H. pylori*, and their use did not provoke the development of resistance [26]. A mixture of *Origanum vulgare* extract with peppermint and *Thymus vulgaris* essential oils showed effects on gut microbiota, potentially improving the composition of the microbiome in pigs [29]. Anethole, flavonoids, and saponins present in peppermint extracts support digestive activity and exert anti-inflammatory effects [28]. The effects associated with *Mentha piperita* extracts and essential oil (EO) can help alleviate symptoms such as heartburn, nausea, bloating, flatulence, and spasms. However, a study on irritable bowel syndrome (IBS) patients did not prove effects of peppermint oil on these symptoms, only pain relief [30]. They can also improve gastric emptying by reducing the feeling of pressure and fullness in the epigastric region, but the study was based on people’s opinions and experiences [28]. A mixture of 8 mL of peppermint oil and Tween 80 (0.2 mL) per 1 L of water plus 0.04% indigo carmine at dosage 200 mL was administered by using a hand pump attached to the accessory channel of the colonoscope. At low doses and short periods, this mixture of peppermint is useful for colon spasm reduction before endoscopy, but higher doses of peppermint can cause heartburn [31].

To reduce bloating, enteric-coated peppermint EO was used three times per day at 180, 360, or 540 mg (10 participants per dose) for a week [27]. EO dosage 0.2 mL or 0.1 mL three times daily in IBS did not prove effective [30]. To achieve antibacterial effects, dosage ranged from 7.8 to 500 mg/mL of EO, depending on the strain of bacteria and type of peppermint [26]. This study does not clarify how the extract was made or the dosing in drops, thus rendering it impossible to recreate the product [25]. This indicates that vast varieties of products are used in studies. Peppermint was used as an EO, extract, dry extract, dried plant material, powder, tincture, and infusion in FSs. These FSs have very wide dosage ranges, such as 20–300 mg or 0.5 mL of peppermint extract, 1.44–6 mg EO, or 180–1200 mg of dried material [20]. Some producers do not describe the preparation or plant part used. An EMA monograph proposes to use 1.5–3 g of herbal material per 100–150 mL three times per and 2–3 mL of tincture three times per day in cases of dyspepsia and flatulence [24]. However, EO is used as herbal medicine, and 0.2–0.4 mL 2–3 times per day for 2 weeks in solid gastro–resistant dosage forms can be used for gastrointestinal issues, especially in the case of IBS [32], meaning that some FS products do not reach active dose used in studies, but some exceed it multiple times. Variable preparations, dosages, and administration routes in studies and FSs make it impossible to compare the effects promised in the health claims of FSs.

Several food supplements containing peppermint include health claims such as “supports healthy digestion,” “helps with indigestion,” and “relieves flatulence and belly spasms.” These claims have been submitted to the EFSA under Article 13.1 of Regulation (EC) No. 1924/2006 as based on generally accepted scientific evidence. However, they have not been authorized. Although these claims are consistent with traditional use and some pharmacological findings, they should not be regarded as scientifically validated. While health claims related to bloating and spasms may be partially supported by individual studies, the overall clinical evidence remains limited.

#### 3.3.2. Artichoke (*Cynara cardunculus* L.)

Artichoke belongs to the *Asteraceae* family. In traditional medicine, artichoke is used to treat functional dyspepsia [33,34]. Artichoke contains biologically active compounds such as cynarin, chlorogenic acid, and phenolic compounds (including flavonoids), as well as minerals, inulin, fiber, and sesquiterpene lactones [34,35]. Inulin, phenolic compounds, and sesquiterpene lactones help reduce oxidative stress and inflammation in the GI tract [33,35]. Polyphenols from artichoke have shown promising bioavailability in vitro [36]. Artichoke was used as dried extract, and 50 µg/mL of artichoke containing preparation was safe and could withstand simulated digestion [34]. Pectins from artichoke (40 and 80 mg/kg) have anti-inflammatory potential in mice with colitis [37]. These properties indicate the artichoke’s ability to relieve symptoms such as pain, dyspepsia, bloating, and indigestion.

Few clinical trials have been conducted on artichoke, most of them regarding antioxidant capacity and hepatic protection and function [38]. Artichoke may also help to reduce symptoms of IBS and dyspeptic syndrome, alleviating abdominal pain, cramps, bloating, flatulence, and constipation [39]. In a different randomized trial, artichoke helped to reduce bloating by improving the microbiota, likely due to its high inulin content [40]. Standardized artichoke leaf extracts 320 or 640 mg daily used for 2 months reduced mild dyspepsia. Although dosage did not influence dyspepsia, patients preferred to use only one capsule per day [41]. Similarly, the dried extract consisting of leaves reduced symptoms in patients with functional dyspepsia when used 320 mg twice per day [42]. Artichoke leaf extract (320 mg for 6 weeks) reduced the symptoms of irritable bowel syndrome [39]. Mixture of 320 mg artichoke and ginger, and 40 mg of simethicone was used to relief belching and bloating in athletes [34].

Artichoke was present in 31 products. The analysis of FSs revealed that artichoke was associated in labeling texts with claims such as promoting and improving digestion and GI health, helping against bloating and GI spasms, and relieving a feeling of heaviness in the abdomen [20]. Most products contained artichoke leaf extract, and doses ranged significantly from 15 to 2500 mg per dosage. Most clinical studies used 320 mg once or twice per day; thus, FS doses under 320 mg might be insufficient to produce any effects.

The EMA has classified artichoke as a traditional herbal medicinal product for the symptomatic relief of digestive disorders, such as dyspepsia accompanied by a sensation of fullness, bloating, and flatulence, based on long-standing use [43]. An EMA monograph describes use of herbal tea 1.5 g with 150 mL with water twice per day and 1500 mg of herbal powder for digestive issues. Dried extract doses ranged from 200 as a single dose to 2700 mg per day, also depending on the extract’s preparation ratio. Again, compared to the dosages used in FSs, it is unlikely that lower doses will have the promised effects. Based on this information, artichoke may help to reduce bloating, pain, cramps, and possibly inflammation, although data is very limited. However, dosage should be considered.

#### 3.3.3. Fennel (*Foeniculum vulgare* Mill.)

Common fennel was present in 29 products. According to the labeling texts, its properties included promoting digestion and gastrointestinal health, improving digestion, helping against bloating and GI spasms, and relieving a feeling of heaviness in the abdomen [20]. Fennel was included in products such as dried plants or powder, various extracts, and essential oil. Daily dosages varied considerably, starting from 6 mg and ranging up to 800 mg.

Fennel is a plant belonging to the parsley family and is traditionally used to prevent various digestive disorders [44,45]. The active substances of fennel found in its EO—especially trans-anethole and fenchone—show promising properties for improving the health of the digestive system [45,46]. It acts both as an antispasmodic agent, relaxing smooth muscles, especially in the GI tract, and as a prokinetic agent, as well as a protective factor for the gastric mucosa in the prevention of ulcers and inflammatory diseases [47]. It also has carminative properties, reducing gas build-up [44].

Fennel exhibits antibacterial properties against *H. pylori* infection (MICs < 50 µg/mL) [48], demonstrating both bacterial-suppressive effects and the ability to protect cells from oxidative stress and reduce inflammatory processes [49,50]. Polyphenols in fennel waste extract also demonstrate antioxidant activity. The same study explored improving the bioavailability of fennel extracts using acid-resistant capsules [51].

Although fennel is used and studied for many applications, in vivo studies in relation to fennel and the digestive system are lacking [44,45]. Chen et al. [47] used 500 g fennel seeds heated in a microwave, wrapped with a towel, and placed on the abdomen close to the skin to reduce postoperative digestion problems. This finding is hard to compare to FSs. The EMA has three entries containing fennel. The indication is based on long-standing use and experimental design studies. *Foeniculum vulgare* Miller subsp. *vulgare* var. *vulgare* therapeutic area is urinary tract and genital disorders, as well as cough and cold. Sweet fennel fruit (*Foeniculum vulgare* Miller subsp. *vulgare* var. *dulce* (Miller)) medicines are traditionally used for symptomatic treatment of mild, spasmodic gastrointestinal complaints, including bloating and flatulence, symptomatic treatment of minor spasm (cramps) associated with menstrual periods, and as an expectorant (helps to expel mucus) in cough associated with colds. For gastrointestinal problems, 1–1.5 g of tea per 100–500 mL of hot water is used. Due to new toxicity studies, the use of EO of fennel is suspended [52]. There is no sufficient data to draw conclusions about the appropriate dosage to support the claims related to FSs.

#### 3.3.4. Milk Thistle (*Silybum marianum* (L.) Gaertn.)

Milk thistle was present in 25 products. The labeling texts of FSs indicate that it supports and promotes digestion and helps to maintain the health of the digestive tract [20]. FS-containing *Milk thistle* had 20–9000 mg of extract or powder per dosage and 5–15 mL of oil used once or several times per day.

Milk thistle is a medicinal plant of the aster family that is used in traditional medicine for the prevention of liver diseases [53,54]. The main active ingredients are silybin A and B and isosilybin A and B, which have been extensively studied as a hepatoprotectants [53]. However, recent studies also reveal its potential in the treatment of other diseases, although research on its effects on digestion-related issues is limited and has mostly been conducted in vitro or in animals [54,55,56]. More research on the impact of Milk thistle on digestion and GI issues has been conducted in veterinary medicine, with several positive outcomes being observed in farm animals [57]. According to the EMA, the fruits of milk thistle are traditionally used as an herbal medicinal product for the symptomatic relief of digestive disorders, including sensations of fullness and indigestion, and to support liver function. It is usually used as tea 3–5 g three times per day, as a powdered herbal substance up to 1800 mg dosed 2–3 times per day, or as various solvent extracts starting at 200 mg daily and ranging up to more than 1000 mg daily. This use is based on traditional experience, provided that serious conditions have been excluded by a medical doctor [58].

Several health claims related to milk thistle have also been submitted to the EFSA, including contributions to liver protection, detoxification, and digestive support. These reflect the plant’s traditional use in supporting both liver and gastrointestinal health.

#### 3.3.5. Dandelion (*Taraxacum officinale* F.H.Wigg.)

There are 21 products registered that contained dandelion. Products contained root, leaf, and inulin, either as dried plant (190–336 mg), extract (50–1000 mg), or dry extract (30–600 mg). The labels indicate properties such as promoting the normal functioning of the stomach, supporting acid–alkaline balance in the stomach, stimulating the release of digestive juices, and exhibiting prebiotic properties that positively affect the functioning of the gastrointestinal tract [20].

Dandelion is an edible medicinal plant distributed worldwide. It is a plant belonging to the aster family and is traditionally used for relieving stomach upset and abdominal pain [59]. Its leaves, flowers, stems, and roots are traditionally used. Dandelion contains several bioactive compounds with gastrointestinal protective properties, including taraxasterol, taraxerol, caffeic acid, chicoric acid, chlorogenic acid, luteolin and its glucosides, polysaccharides, inulin, and β-sitosterol. These dandelion-derived substances demonstrate potential pharmacological efficacy in the treatment and management of various gastrointestinal disorders, including dyspepsia, gastroesophageal reflux disease, gastritis and *H. pylori*-associated gastritis, small-intestinal ulcers, ulcerative colitis, gastrointestinal malignancies, liver diseases, gallstones, and acute pancreatitis [60]. Although the review includes valuable insights, some of the sources cited are informational books intended for the general public rather than peer-reviewed scientific publications. Therefore, while the findings are informative, they highlight the need for more rigorous clinical trials and scientifically validated data in the future.

In the study, dandelion’s aerial part shows anti-inflammatory, antioxidant, and protective properties for gastric mucosa. Dandelion leaf extracts have demonstrated the ability to stabilize the pH of the gastric environment and to relieve inflammation of the mucous membrane. The biologically active substances contained in the extracts include sesquiterpene lactones, which have anti-inflammatory and antispasmodic effects; flavonoids and phenolic acids (e.g., chlorogenic acid), which exhibit antioxidant activity; triterpenoids, steroids, and coumarins, which show anti-inflammatory and immunomodulatory properties; and saponins, which protect the gastric mucosa against damage caused by stomach acid [59]. In the study by [59], 30 or 300 mg/kg of dried extract of dandelion aerial parts were used on mice, and they showed a significant reduction in the size of the injured area. Chicoric acid was the main component identified in dandelion aerial-part extracts in this in vitro study (in human colonic epithelial cell line HT-29). They provided anti-inflammatory and antioxidative effects in cases of inflammatory bowel disease and colorectal cancer [61]. The effect on *H. pylori* was tested in this study, and chicoric acid and luteonin in dandelion flower extract showed promising antibacterial effects [62].

According to EMA monographs, various parts of dandelion are traditionally used as herbal medicinal products for several indications. Dandelion root is traditionally employed for the relief of symptoms associated with mild digestive disorders, including the sensation of abdominal fullness, flatulence, and slow digestion, as well as for temporary loss of appetite. Additionally, it is used to increase urine output to promote flushing of the urinary tract as an adjuvant in minor urinary complaints [63]. Preparations containing both dandelion root and herb share similar traditional uses, serving to alleviate mild digestive disturbances and temporary loss of appetite while also supporting urinary tract health through diuretic effects [64]. Dandelion leaf, likewise, is traditionally used to increase the amount of urine for urinary tract flushing as an adjunct treatment in minor urinary complaints. Preparations from root include decoction 1–5 g 2–3 times daily, juice 4–8 mL, extract 2–8 mL, or tincture 5–10 mL three times daily. Root and herb monograph notes the use of stronger decoction (3–4 g three times per day), dry extract tablets 150–300 mg 2–3 times daily, and liquid extracts and juice. These uses are based on long-standing traditional experience rather than established clinical evidence [65].

Furthermore, several health claims related to dandelion have been submitted to the EFSA, including its contribution to liver and gallbladder function, digestive comfort, maintenance of acid-base balance, and prebiotic effects that support GI well-being. While these claims reflect the plant’s traditional applications, the available scientific evidence remains limited. Studies have been conducted on various plant parts, extract types, and dosages, which do not always correspond to those used in food supplements. Due to the limited number of high-quality clinical studies, it is challenging to fully verify the accuracy of the health claims presented on FS labels. Nonetheless, dandelion extracts show potential as therapeutic agents in conditions such as gastritis, GERD, and GI inflammation, warranting further investigation.

#### 3.3.6. Chamomile (*Matricaria recutita* L.)

Chamomile was included in 19 products, with labeling texts indicating properties such as promoting digestion and GI tract health, improving digestion and gastric juice secretion, promoting regular GI motility and gas elimination, relieving bloating, and preventing spasms. Products contained 8–600 mg extract or powders 200–525 mg; some products were dosed by mL (0.2–0.6). All FSs used the flowers of chamomile [20].

Chamomile belongs to the aster family and has traditionally been used to treat the symptoms of elevated stomach acid, as well as for its antispasmodic and anti-inflammatory properties [22,25]. Chamomile is a widely used plant and has been the subject of many reviews regarding its biological activities [66]. The main biologically active substances in chamomile include apigenin, bisabolol, chamazulene, flavonoids, tannins, alkaloids, glycosides, and saponins. Bisabolol and chamazulene exhibit antibacterial effects against *H. pylori* and possess antioxidant activity. Flavonoids such as quercetin and patuletin act as antioxidants, protecting the mucous membranes of the GI tract. Moreover, tannins, alkaloids, glycosides, and saponins demonstrate anti-inflammatory and anti-ulcerative properties [66,67]. Apigenin is a powerful anti-inflammatory antispasmodic agent [68]. Furthermore, the EO of chamomile has antibacterial effects against *H. pylori* [66,67]. Chamomile can also improve the healing process of peptic ulcers [67]. Chamomile in combination with peppermint extract reduced GI discomfort and pain [25]. An analysis of studies on herbal medicinal product Iberogast (contains 66.7 mg of chamomile) reports reduced indigestion symptoms and treatment of functional dyspepsia [69]. Thus, chamomile is a promising gastroprotective that reduces stomach spasm, flatulence, stomachache, and decreases gastric secretion.

According to the European Union herbal monograph on *Matricaria recutita* L., *flos*, chamomile flower is traditionally used as an herbal medicinal product for several indications. It is utilized for the symptomatic treatment of minor GI complaints, such as bloating and minor spasms, as well as for the relief of the symptoms associated with the common cold. Additionally, chamomile flower is used for the treatment of minor ulcers and inflammations of the mouth and throat and as adjuvant therapy for irritations of the skin and mucosae in the anal and genital regions, provided that serious conditions have been excluded by a medical doctor. It is also traditionally used for the treatment of minor skin inflammations, such as sunburn, superficial wounds, and small boils (furuncles) [70]. In cases of indigestion, 100–150 mL of an infusion containing 1–4 g of herbal substance is used three times daily, or a 70% ethanol-based liquid extract 1–4 mL three times daily. Furthermore, chamomile EO is used as a traditional herbal medicinal product for adjuvant therapy of irritations of the skin and mucosae in the anal and genital regions under similar precautionary conditions [71]. There are substantial differences in preparations and dosages between FSs, studies, and EMA monographs.

Furthermore, several health claims related to chamomile have been submitted to the EFSA, including support for the treatment of GI complaints such as minor spasms, epigastric distension, flatulence, belching, and indigestion. These claims are consistent with both the plant’s traditional use and its known pharmacological properties. Chamomile’s antispasmodic, anti-inflammatory, and antibacterial effects, particularly against *H. pylori*, suggest its potential role in relieving symptoms related to increased gastric acid, gastritis, and functional GI disorders. While additional clinical studies are needed to fully substantiate these claims, the currently available evidence supports the use of chamomile as a promising herbal candidate for alleviating mild digestive discomfort.

#### 3.3.7. Blond Psyllium (*Plantago ovata* Phil.)

*Plantago ovata* (*Plantago australis* subsp. *cumingiana* (Fisch. & C.A.Mey.) Rahn) was present in 18 FS compositions. Most products contained powdered seed husks with doses ranging from 1490 to 6990 mg. The labels indicate that it may help to maintain normal digestive system function and facilitate and improve digestion [20].

Plantain (psyllium), a plant belonging to the plantain family (*Plantaginaceae*), has been traditionally used in cases of indigestion, inflammation, constipation, and for the prevention of generalized abdominal pain. Its seeds and husk are known as a source of mucilaginous fiber that forms a gel when mixed with water. Psyllium is used in the treatment of constipation and for the prevention or management of conditions such as Crohn’s disease-related constipation, diarrhea, obesity, hypercholesterolemia, diabetes, and atherosclerosis [72,73]. Dosages mentioned in studies include 5 g in 250 mL three times daily [74] and 12 g of psyllium in 250 mL water [72,75]. It also may have protective effects on intestinal mucosa and protect against gastric ulcers [73,76].

According to EMA monographs, ispaghula husk and ispaghula seed derived from Plantago ovata have well-established uses as herbal medicinal products, primarily for the treatment of habitual constipation. They are also indicated in conditions where easy defecation with soft stool is desirable, such as in cases of painful defecation following rectal or anal surgery, anal fissures, and hemorrhoids. Additionally, ispaghula husk is recommended for patients requiring increased daily fiber intake, for example, as an adjuvant treatment in constipation-predominant irritable bowel syndrome and as a dietary adjunct in managing hypercholesterolemia. It is essential that sufficient liquid is consumed alongside ispaghula products—for instance, approximately 30 mL of water per 1 g of herbal substance—to ensure safety and efficacy [77,78]. Comparing the dosage in products with studies, fiber intake from FSs would be too low in a single dose and should be increased and used for longer periods of time.

Psyllium has demonstrated effectiveness in supporting GI health, particularly through its high fiber content that aids intestinal transit and stool regularity. The EFSA-submitted health claims for psyllium include its contribution to intestinal function, stool softening, and bowel regularity, as well as its role in maintaining healthy cholesterol levels. These claims align with both traditional uses and clinical evidence supporting psyllium’s physiological effects. However, some labeling terms, such as “facilitates digestion” or “improves digestion”, remain ambiguous and are difficult to substantiate directly based on the scientific literature. Therefore, while the functional benefits related to fiber content are well supported, broader digestive claims should be interpreted cautiously.

#### 3.3.8. Caraway (*Carum carvi* L.)

Caraway was present in 11 FS products, with labels indicating properties such as supporting digestive system function, stimulating digestion, reducing gas accumulation in the GI tract, and alleviating the feeling of heaviness in the abdomen. FSs contained four different preparation types: 90 mg seed of caraway, 100–200 mg extract, powdered seeds 150–500 mg, and oil 0.45 mL. FSs contained four different preparation types: 90 mg seed of caraway, 100–200 mg extract, powdered seeds 150–500 mg, and oil 0.45 mL [20].

Caraway is a medicinal plant belonging to the parsley family and is traditionally used for conditions such as increased stomach acid, burning sensations in the epigastrium, and various stomach and digestive disorders [22,79]. However, Šarić-Kundalić et al.’s [79] study is a survey of patients’ experiences. Studies on humans show that it can reduce gastrointestinal discomfort, dyspeptic symptoms, and spasms, and in vitro, it had an effect on *H. pylori* [80].

Caraway EO has known antibacterial effects and contains carvone and limonene [81]. The EO also contains carveol (p-ment-6,8-dien-2-ol), which exhibits anti-inflammatory, antisecretory, antioxidant, and antimicrobial properties, while 1,8-cineol demonstrates anti-inflammatory and GI mucosal protective effects. Carveol possesses antisecretory activity by reducing gastric acid secretion and maintaining physiological pH levels. It also exerts cytoprotective effects by promoting the protection and healing of gastric mucosal cells and functions as an antioxidant and immunomodulator, alleviating oxidative stress and inflammatory processes in the gastric mucosa [82]. It has demonstrated H+/K + -ATPase pathway inhibition, inhibiting gastric acid secretion, and reducing gastric spasms in *H. pylori* infection [83]. Caraway seed extract contains also phenolic compounds that have antioxidant activities [84]. Conversely, studies were performed in vitro or animals.

A study investigating the impact of ginger (1 g), caraway (2 g), and peppermint (3 mL) daily on gastrointestinal complaints—including postprandial distress, early satiation, epigastric pain, epigastric burning, nausea, vomiting, and belching—in tuberculosis patients revealed that nausea, vomiting, bloating, and abdominal pain were reduced by these plants. The reduction in pain was primarily attributed to caraway [85].

In general, the EMA considers caraway fruit and essential oil to be safe and effective for reducing gastrointestinal discomforts such as bloating and flatulence, based on long-standing use in traditional medicine [86]. In the caraway’s monographs 0.5–2 g of the herbal substance in 150 mL in infusion of seed or 0.15–0.3 mL of the essential oil divided in 1–3 doses daily is described for digestive issues [86,87].

Caraway shows potential in managing excess gastric acid through its antisecretory and mucosa-protective effects. These properties support its traditional use and align with health claims seen on food supplements, although more targeted clinical evidence is needed. Additionally, most of the FSs contained rather low concentrations of caraway or its extracts which could impact effect of production in relation to health claims.

#### 3.3.9. Liquorice (*Glycyrrhiza glabra* L.)

Liquorice was mentioned in 12 FS products, which included extracts of 10–200 mg root and rhizome; only 1 product contained 100 mg of dried plant material. According to the labels, it helps to maintain normal digestive system function, supports the digestive process and immune system function, and possesses antioxidant properties [20].

The *Glycyrrhiza* genus encompasses over 30 species that are widely distributed across the globe. Historically, liquorice was a highly valued medicinal herb in Ancient Egyptian, Roman, Greek, East Asian, and Western cultures. Licorice root extracts offer numerous health benefits, including the treatment of throat infections, tuberculosis, and respiratory and liver diseases, and also possess antibacterial, anti-inflammatory, antioxidant, and immunomodulatory properties [88]. Its main components include licorice saponins (glycyrrhizinic acid), flavonoids, other phenolic compounds, polysaccharides, and essential oils [89].

This review provides extensive data on liquorice, reporting its effects in several clinical trials. However, no significant difference was observed between placebo and liquorice in the treatment of gastric ulcers (dosage of 2 g aqueous liquorice root extract or 500 mg deglycyrrhizinised liquorice). Other studies mentioned in the review did not report positive outcomes or any significant effects related to GI issues. Nevertheless, the addition of liquorice to clarithromycin therapy improved *H. pylori* eradication rates [88]. An Indian product, Gutgard, containing 150 mg of licorice, showed positive outcomes in cases of *H. pylori* treatment [90]. A recent review reports on the possible use of licorice against gastric cancer [89]. Novel delivery systems for liquorice extracts have been reported, including carbon dots, which have shown a promising anti-ulcer effects in mouse models of acute alcoholic gastric ulcer [91]. Plant extracts from *Trifolium pratense*, *Glycyrrhiza glabra*, and Myristica fragrans encapsulated in an alginate microcapsule emulsion demonstrated antibacterial activity in vitro [92].

EMA also supports its use in relieving burning sensations and dyspepsia. Liquorice should be used as tea 3–8 g daily or as extract 64–96 mg (max 160 mg) daily for two weeks [93]. Although some positive reports are available regarding the effects of licorice on GI issues, these benefits are mostly related to its antibacterial activity and ulcer treatment. However, liquorice should be used cautiously in individuals with cardiac conditions and by those taking medications such as diuretics, cardiac glycosides, corticosteroids, stimulants, laxatives, or other drugs that may exacerbate electrolyte imbalances [93].

FSs contain differing extracts than clinical trials, and some even exceeded the EMA’s suggested dosage, although it is unknown what type of extract is used in these supplements. FSs containing liquorice should be used with caution.

#### 3.3.10. Chicory (*Cichorium intybus* L.)

Chicory was included in the composition of 12 FSs, with label texts indicating properties such as helping to maintain normal digestive function, supporting digestion, and promoting the release of digestive juices [20]. Most of products had powdered chicory root with broad-ranging dosages of 45–5000 mg.

Chicory is a plant belonging to the aster family (*Asteraceae*). Traditionally, infusions of chicory root have been used to alleviate symptoms of nausea and increased stomach acid [22]. According to the Community herbal monograph on *Cichorium intybus* L., *radix*, chicory is classified as a traditional herbal medicinal product for the relief of symptoms related to mild digestive disorders, such as a feeling of abdominal fullness, flatulence, and slow digestion, as well as for temporary loss of appetite [94]. Its use dates back centuries; however, it is supported by traditional knowledge rather than evidence from clinical trials [94,95].

The main components of its roots are chicoric acid, sesquiterpene lactones, and guaianolides, which contribute to its bitter taste [94]. The root of this plant is a valuable prebiotic and gastroprotective agent due to its high content of inulin, chlorogenic acid, and sesquiterpenes [96,97]. It can protect the gastric mucosa, reduce inflammatory processes and oxidative stress, and improve the balance of intestinal microflora and GI motility. Additionally, since it contains fiber, it contributes to the overall health of the digestive system [97,98]. Pouille et al. [97] prepared chicory extract from 3 g of root in 150 mL if water and used 2 g of this freeze-dried extract was used in simulated gastric tract and concluded that chicory remained it antioxidant and inflammatory properties. Chicory exhibits very low cytotoxicity and is therefore considered safe for use [96]. The EFSA-submitted health claims align well with traditional uses and pharmacological data on chicory root, which highlight its prebiotic, bitter, and motility-enhancing properties. Similarly to dandelion, chicory may support digestion through stimulation of bile secretion, improved gut motility, and modulation of the intestinal microbiota. However, the dosage has to be high to achieve these positive effects.

#### 3.3.11. Lemon Balm (*Melissa officinalis* L.)

Lemon balm was also mentioned in the labels of 11 FS products, with health claims indicating that it promotes digestion, supports regular GI motility, helps to reduce gas leading to bloating, and has a positive impact in cases of indigestion and feelings of heaviness [20]. Most FSs had extract of leaf (11–300 mg); one was a tincture containing 3.9 mg of leaf.

Lemon balm is an aromatic and edible plant belonging to the mint family and has been extensively studied due to its broad medicinal properties, including antioxidant, antimicrobial, antidepressant, anti-inflammatory, and antiangiogenic activities [99]. According to the EMA, Lemon balm is classified as a traditional herbal medicinal product for the relief of mild symptoms of mental stress and to aid sleep, as well as for the symptomatic treatment of mild gastrointestinal complaints, including bloating and flatulence. For preparation of 150 mL of infusion 1.5–4.5 g of herbal substance is used. Other products are as follows: 2–6 mL tincture; 2–4 mL liquid extract; 0.19–0.55 g powdered herbal substance: dried water or ethanol (45–53% *V*/*V*). All are used 1–3 times daily for two weeks [100]. In traditional medicine, it is used for conditions such as increased stomach acid and generalized abdominal pain [15,22,101], but little is known about its effects and mechanisms of action in the GI tract [102]. Reports indicate that it has antispasmodic effects, relieves bloating and gas formation, and exhibits gastroprotective properties. It may reduce the formation of stomach ulcers, increase the activity of antioxidant enzymes, decrease lipid peroxidation, and accelerate gastric emptying, thereby improving digestion [99,101,102]. Lemon balm extract exhibits dose-dependent and site-specific effects within the GI tract; in particular, it can influence motility in the ileum and jejunum ex vivo [102]. Lemon balm extract demonstrated a dose-dependent protective effect against gastric ulcers in mice, comparable to that of omeprazole, which strongly correlated with its antioxidant activity [103]. In mixtures with fennel and chamomile, it reduced colic symptoms in infants [104]. Thus, doses of FSs should be assessed before use.

Its ethanolic extracts contain tannins, flavonoids, and phenolic compounds [103]. The main biologically active substances in extracts of this plant include rosmarinic acid; flavonoids such as luteolin; and phenolic acids like caffeic acid and salicylic acid [99]. These effects are associated with both essential oil components and phenolic compounds.

The EFSA-submitted claim for lemon balm refers broadly to its support of digestion and intestinal function, but the wording remains general and lacks specificity regarding its physiological mechanisms. While this aligns with traditional use and some pharmacological data, such a generalized statement provides limited clarity in assessing the substantiation of health claims related to digestive effects

## 4. Discussion

The results of this study highlight the therapeutic potential of these medicinal plants for the development of new phytopharmaceuticals. A comparison of the identified health claims and their supporting evidence is provided in Table S1.

The health claims on labels are only supported by the traditional use of plants. Although claims on products were similar to those in EFSA claim registry, all of these claims are on hold due to lack of evidence. Additional EMA monographs are also based only on the long-standing use of plants. Even such popular plants as peppermint, chamomile, fennel, and caraway, which are frequently used in home remedies, lack substantial evidence that is based on randomized controlled trials. Effects on indigestion are reported in surveys for peppermint [29] and caraway [79]. Some claims are very generic, such as “helping maintain normal functions of GI”. Thus, it is hard to test the claim in studies or models. Detailed study design reveals specific properties of the plant extract [41,85]. Another difficulty in reaching conclusions from studies with positive outcomes is the use of combined formulation [69,101]. Although this herbal medicine has good evidence, data can hardly be transferred to other FS products [69]. An additional problem is the specific study design in this trial [47], which used heated seeds on the stomach that are incomparable to FSs. Most studies reveal promising results for plants in vitro, such as antioxidants, anti-inflammatory, and antibacterial effects against *H. pylori* [48,49,50,61,62,66,67,81,84,96]. Dandelion [59] and caraway [80] have the potential to reduce gastric acid and stabilize pH in the stomach. Lemon balm [99,101,102], peppermint [27,28,29,30,31], and caraway [80] have promising results in vitro and animal models regarding spasm and pain reduction. Additionally, there are reports of reduction in flatulence, bloating, discomfort, and dyspepsia for peppermint [25], artichoke [34,39,40,41], chamomile [69], psyllium [72], caraway [80], and Lemon balm [101,102,103,104].

However, the current clinical evidence, particularly from human trials, remains limited. Additional research is required, especially from well-designed in vitro and in vivo studies, to clarify potential side effects, determine safe and therapeutically effective dosages, and evaluate efficacy in comparison to conventional treatments such as proton pump inhibitors (PPIs). Other studies have also emphasized the scarcity of robust data supporting the effectiveness of these plants in improving GI function or alleviating GI disorders [105]. Other authors emphasize that the results of plant-related clinical trials are difficult to compare due to their heterogeneity [106] and quality [107]. Lack of herbal product standardization also is cause for concern. Only some studies stated the standardization of their extract [41,42].

Regarding FSs, consumers must interpret the information on product labels with great caution. FSs often differ from traditional herbal medicines and clinical trial formulations in terms of plant parts used, extract types, and dosages [9,12]. In this context, EMA monographs for traditional herbal medicinal products provide authoritative summaries of well-established and traditional uses, quality standards, and recommended indications. However, many FS claims only partially align with these EMA monographs, as they often lack equivalent evidence or standardization. For example, peppermint essential oil has good clinical evidence for use in particular GI problems and has herbal medicinal products, but the data is not applicable to other peppermint extracts or FSs, since they have different health claims and formulations [108].

Our analysis shows that researchers various plant preparations such as dried herbal material, extracts, oils, EO. Additionally, their extraction modes, solvents, and ratios of plant to extractant differ significantly. Similarly, FS producers use a broad range of preparations and dosage forms: capsules, tablets, tinctures, solutions, syrups, and tea. Some FS manufacturers do not describe plant or parts used in products, but only state the name and weight of the plant, thus making comparisons with research or monographs difficult. In addition, studies note that plant active compounds can be modified and neutralized in the gastric tract; thus, they need to be protected by modified tablets or capsules [51]. Also, the effects could be dose-dependent [103]; therefore, this should be considered before assigning health claims.

Moreover, each study used different amounts of extract at various time intervals and durations. Plant monographs also allow for the use of herbal tea, powders, and extracts of different concentrations. For these uses, each therapeutic indication has assigned posology. In addition, all doses of FSs for one plant differed, sometimes between tens and hundreds of times. Consequently, the outcomes of clinical trials cannot be transferred or should be applied with caution to the health claims of FSs due to major posology differences.

In summary, medicinal plants with acid-modulating and GI-protective properties present promising adjunct or preventive options for GI disorders. Nevertheless, to ensure their safe and effective application, further research is essential to develop standardized preparations, dosage forms, and evidence-based guidelines.

Importantly, under the EU regulatory framework, health claims for botanicals remain a special and unresolved category. Many claims submitted to EFSA under Article 13.1 of Regulation (EC) No. 1924/2006 have been placed “on hold,” meaning they are neither officially authorized nor rejected. The European Commission, following EFSA’s scientific opinion, has the final decision-making role. Under transitional provisions, such claims may still appear on products in EU Member States, provided that they are not misleading and comply with national enforcement practices. This situation places much of the responsibility for substantiation on manufacturers and illustrates the lack of harmonization in the regulation of botanical health claims across the EU. The unclear status of health claims misleads the population as it is not forbidden to use unsupported claims. EU Member States differ on how to interpret and implement these regulations, causing inconsistency in the market and confusion among both businesses and consumers. There is ongoing debate about whether to allow for a graded approach to evidence, where traditional use could be considered lower-grade support for claims, but this is not currently accepted legally. This stresses the need for finalizing status of health claims and more rigorous implementation of regulations. Moreover, on 30 April 2025, the Court of Justice of the European Union (CJEU) ruled that food products containing botanical ingredients may use health claims only if they have been authorized by the European Commission following an EFSA evaluation (Novel Nutriology, case C-386/23). Claims covered by the transitional provisions of the EU Health Claims Regulation remain exempt. This judgment clarifies earlier legal uncertainty and confirms that only claims listed in Regulation (EU) No. 432/2012 or those under transitional rules are permitted.

This study provides new insights by systematically analyzing the use of medicinal plants specifically in FSs for GI health. It identifies the most frequently used plants in FSs, many of which have not been thoroughly studied in clinical settings. By comparing labeling claims with scientific evidence, this work fills a knowledge gap and enhances our understanding of the real-world application of medicinal plants in GI health management.

Although our analysis focused on the Latvian market, the FS producers included both local and international companies operating across the EU and other regions. Botanical health claims are permitted in all countries, and similar claims were identified in different markets when identical products were searched online. Since food supplements and herbal teas are easily accessible through online platforms that deliver worldwide, label information is relevant to a broad audience. Therefore, the analysis of plants presented in this review may be applicable to many countries.

Dataset [20] provides a compilation of the plants commonly used in FSs, including the plant part, preparation type, dosage, and associated health claim. This information can be used in future studies and in planning research to test these claims. It also offers healthcare professionals data to support decision-making when consulting patients about FS use. The dataset could be further expanded by adding other FSs, and additional plant health claims could be analyzed more easily.

From a public health and regulatory perspective, these findings have practical relevance. They promote consumer awareness about the limitations of health claims on FS labels and highlight the importance of critical interpretation. The study also supports evidence-based decision-making by consumers, healthcare providers, and policymakers, fostering a safer and more informed use of plant-based supplements.

Finally, this study excluded plants found in fewer than ten FSs. Future research could expand the analysis to include less commonly used species, such as those from the parsley (*Apiaceae*) or aster (*Asteraceae*) families, many of which hold potential based on traditional use, including examples such as *Arctium lappa* (great burdock), *Aloe* spp., or *Salvia Officinalis* (Sage). Future studies could analyze commonly used plants from other parts of the world, provide comparison of their use in different cultures. This could be useful for the harmonization of plant use globally.

## 5. Conclusions

This study highlights that, although plants such as peppermint, caraway, chamomile, lemon balm, dandelion, licorice, artichoke, chicory, fennel, milk thistle, and psyllium are frequently included in food supplements with digestive health claims, many of the statements found on product labels are not sufficiently supported by clinical evidence. This reveals significant regulatory gaps and underscores the need for the harmonized evaluation of plant-based health claims to ensure their scientific validity and consumer clarity.

## Figures and Tables

**Table 1:** Most commonly used plants in FSs in Latvia.

Plant	Product Mentions
*Mentha piperita*	35
*Cynara scolymus*, syn. *Cynara cardunculus*	31
*Zingiber officinale*	29
*Foeniculum vulgare*, and *F. vulgare var. dulce*	29
*Silybum marianum*	25
*Piper nigrum*	21
*Taraxacum officinale*	21
*Matricaria chamomilla*	19
*Curcuma longa*	18
*Plantago ovata*	18
*Emblica officinalis*	15
*Aloe* spp.	12
*Cichorium intybus*	12
*Glycyrrhiza glabra*	12
*Carum carvi*	11
*Melissa officinalis*	11

**Table 2:** Plants used for digestive tract issues in Eastern and Central Europe.

Family	English Plant Name	Latin Plant Name
Rose (*Rosaceae*)	*Agrimony*	*Agrimonia eupatoria* L.
	Cinquefoils and tormentil	*Potentilla* L. spp.
Mallows (*Malvaceae*)	Marshmallow	*Althaea officinalis* L.
	Common mallow	*Malva sylvestris* L.
Aster (*Asteraceae*)	Wormwood	*Artemisia absinthium* L.
	Chicory	*Cichorium intybus* L.
	Roman Chamomile	*Matricaria chamomilla* L. syn. *Chamomilla chamomilla* (L.) Rydb. *Chamomilla recutita* (L.) *Rauschert* *Matricaria recutita* L.
Parsley *(Apiaceae)*	Caraway	*Carum carvi* L.
	Fennel	*Foeniculum vulgare* Mill.
	Parsley	*Petroselinum crispum* (Mill.) Fuss
Gentian *(Gentianaceae)*	Yellow gentian	*Gentiana lutea* L.
Conifers *(Cupressaceae)*	Juniper	*Juniperus communis* L.
Mint *(Lamiaceae)*	Horehound	*Marrubium vulgare* L.
	Melissa	*Melissa officinalis* L.
	Mints	*Mentha* L. spp.
Ferns *(Polypodiaceae)*	Polypody	*Polypodium vulgare* L.

**Table 4:** Types of preparation included in the herbal products.

Preparation Type	Count
Extract	239
Powder	131
Dry extract	63
Dried plant	32
Essential oil	13
Oil	11
Juice	7
Tincture	3
Juice concentrate	3
Ethyl alcohol extract	2
Gel	2
Pellets	2
Fiber	2
Leaf	2
Liquid extract	2
Powder, Dry extract	1
Leaf powder, freeze-dried fruit	1
Squeezes	1
Extract concentrate	1
Dry extract, Essential oil	1
Seed	1
Dry juice	1
Inulin	1
Oil concentrate	1
Infusion	1
not mentioned	9

**Table 3:** Plants used for generalized abdominal pain and used for sharp throbbing abdominal pain.

Family	English Plant Name	Latin Plant Name
Parsley *(Apiaceae)*	Lovage	*Levisticum officinale* W.D.J. Koch.
	Burnet-saxifrage	*Pimpinella* L. spp.
Lilly *(Asphodelaceae)*	Aloe	*Aloe* sp. L.
Aster *(Asteraceae)*	Elecampane	*Inula helenium* L.
	Southernwood	*Artemisia abrotanum* L.
	Spear thistle	*Cirsium vulgare* (Savi) Ten.
	Cornflower	*Centaurea cyanus* L.
Birch *(Betulaceae)*	Birch	*Betula* L.
Carnation *(Caryophyllaceae)*	Maiden pink	*Dianthus deltoides* L.
	Rupturewort	*Herniaria* Tourn. ex L.
	Sticky catchfly	*Viscaria vulgaris* Bernh.
Conifers *(Cupressaceae)*	Juniper	*Juniperus communis* L.
Heath *(Ericaceae)*	Partridgeberry	*Vaccinium vitis-idaea* L.
Geranium *(Geraniaceae)*	Pelargonium	*Pelargonium* L’Her.
Grasses *(Poaceae)*	Couch grass	*Agropyron* Gaertn.
Buttercup *(Ranunculaceae)*	Aconite	*Aconitum napellus* L.
	Field larkspur	*Consolida regalis* Gray
Rose *(Rosaceae)*	Cinquefoil	*Potentilla* L.
	Silver weed	*Potentilla anserina* L.
Nightshade *(Solanaceae)*	Jimsonweed	*Datura stramonium* L.
	Henbane	*Hyoscyamus niger* L.
Nettle *(Urticaceae)*	Nettle	*Urtica* L.

## Data Availability

The data presented in this study are openly available in the Rīga Stradiņš University Institutional Repository Dataverse at https://doi.org/10.48510/FK2/4YKBAS. Reference: Teterovska, R., Skotele, R. E., Maurina, B., and Sile, I. (2025). ‘Analysis of the botanical composition and health claims of food supplements for gastrointestinal disorders’. Version 1. Rīga Stradiņš University [20].

## Supplementary Materials

The following supporting information can be downloaded at https://www.mdpi.com/article/10.3390/nu17233674/s1. Table S1: Comparison of the identified health claims and their supporting evidence.

## Abbreviations

The following abbreviations are used in this manuscript:

GI	Gastrointestinal
EMA	European Medicines Agency
EFSA	European Food Safety Authority
PPIs	Proton Pump Inhibitors
FS	Food Supplement
EO	Essential Oil
GERD	Gastroesophageal Reflux Disease
HMP	Herbal Medicinal Product
PVD	Food Supplements Register (in Latvian: Pārtikas un Veterinārais Dienests)
EU	European Union
