# Supplementary material for: Medicinal Plants in Food Supplements for Gastrointestinal Disorders: Critical Assessment of Health Claims on Gastric Acid Regulation
Source: Nutrients. 2025 Nov 24;17(23):3674. doi: 10.3390/nu17233674 (PMC12693992; doi:10.3390/nu17233674)
Supplement: Supplementary file 1 [file nutrients-17-03674-s001.zip › nutrients-3975920-SM final.pdf]

Table S1: Comparison of the identified health claims and their supporting evidence

| *Plant* | *Claims on FS* | *EMA* | *EFSA* | *Evidence in Studies* | *Evidence Level* |
|---|---|---|---|---|---|
| *Peppermint extract* | Beneficial effect on the functioning of the digestive system, contributes to normal GI function, improves stomach function, stimulates digestion, and prevents flatulence and abdominal cramps | Relief of digestion-related problems, such as indigestion and flatulence<br>LSTU | Helps to support a healthy digestion<br>Has a positive influence on intestinal health<br>Contributes to digestive functions<br>Contributes to the normal function of intestinal tract<br>Helps keep the stomach healthy<br>Helps with flatulence and belly spasm<br>Helps with indigestion<br>"On hold" status | Trial [25]<br>Patients in ICU—flatulence | Low evidence<br>Flatulence, but not applicable to general population |
| *Peppermint essential oil* | Beneficial effect on the digestive system, promoting normal functioning of the GI tract | Herbal medicinal product for the symptomatic relief of minor spasms of the GI tract, flatulence and abdominal pain, especially in patients with irritable bowel syndrome<br>Well-established use | Not authorised | Trial [27]: children, antispasmodic effects<br>Trial [30]: patients with IBS—no effect, only pain relief<br>Clinical trial [31]: coloscopy procedure spasm relief<br>Questionnaire [28]: people's experience with feeling of pressure and fullness | Good evidence<br>Antispasmodic |
| *Artichoke* | Promoting and improving digestion and GI health, helps against bloating and GI spasms, | Symptomatic relief of digestive disorders, such as dyspepsia accompanied by a sensation of fullness, bloating, and flatulence | Helps to support digestion<br>Contributes to the normal function of intestinal tract<br>Contributes to intestinal comfort | Trial [39]: dyspeptic syndrome and IBS<br>Randomized trial [40]: reduces bloating | Low evidence<br>Possibly bloating, dyspeptic symptoms |

Table S1: Comparison of the identified health claims and their supporting evidence

| | | | | | |
|---|---|---|---|---|---|
| | and relieves a feeling of heaviness in the abdomen | LSTU | Contributes to the normal function of intestinal tract<br>Support of detoxification<br>Helps support the digestive juice flow<br>Maintains a healthy liver<br>Contributes to intestinal comfort<br>"On hold" | Trial [41]: people with mild dyspepsia—reduction in some symptoms<br>Trial [34]: belching and bloating in athletes | |
| *Fennel* | Promotes digestion and GI health, improves digestion, helps against bloating and GI spasms, and relieves a feeling of heaviness in the abdomen | For sweet fennel: symptomatic treatment of mild, spasmodic GI complaints including bloating and flatulence<br>LSTU | Supports appetite, digestion and elimination<br>Supports the health of the digestive tract<br>Helps with flatulence and belly spasm<br>Helps to support digestion<br>"On hold" | Trial [47], with heated seeds on stomach<br>In vitro [48]: antibacterial *H. pylori*<br>In vitro [49,50]: anti-inflammatory, antioxidant | Very low evidence<br>Possibly antibacterial, anti-inflammatory, bloating |
| *Milk thistle* | Supports and promotes digestion and helps maintain the health of the digestive tract | Symptomatic relief of digestive disorders, including sensations of fullness and indigestion, and to support liver function<br>LSTU | Supports liver health<br>Contributes to liver protection<br>Contributes to the detoxifying potential of the liver<br>Helps to maintain the liver function and additionally promote the digestion and the body´s purification<br>"On-hold" | In vitro or in animals [54–56]: digestive improvement<br>Farm animal studies [57] | Very low evidence<br>Possibly improves digestion |
| *Dandelion* | Promotes the normal functioning of the | Relief of symptoms associated with mild digestive disorders, | Improves liver and gallbladder functions | In vivo on mice [59]: stabilized the pH of the gastric environment and relieved | Very low |

Table S1: Comparison of the identified health claims and their supporting evidence

| | | | | | |
|---|---|---|---|---|---|
| | stomach, supporting acid-alkaline balance in the stomach, stimulates the release of digestive juices, and exhibits prebiotic properties that positively affect the functioning of the GI tract. | including a sensation of abdominal fullness, flatulence, and slow digestion, as well as for temporary loss of appetite<br>LSTU | Supports the liver and biliary functions<br>Promotes the digestive comfort<br>Helps to facilitate fat digestion<br>Contributes to the functions of the intestinal tract<br>Helps the physiological pH balance of the stomach<br>Stimulates the digestion<br>"On hold" | inflammation of the mucous membrane<br>In vitro [61]: anti-inflammatory and antioxidative effects<br>In vitro [62]: antibacterial *H. pylori* | Possibly stabilizes pH, and is antibacterial |
| *Chamomile* | Promotes digestion and GI tract health, improves digestion and gastric juice secretion, promotes regular GI motility and gas elimination, relieves bloating, and prevents spasms | Bloating and minor spasms<br>LSTU | Helps to supports the treatment of gastro-intestinal complaints such as minor spasms epigastric distension, flatulence and belching<br>Helps with indigestion and flatulence<br>"On hold" | In vitro [66,67]: antibacterial *H. pylori*<br>In vivo [67]: rats with GI lesions<br>Trial [69]: reduced indigestion symptoms and treatment of functional dyspepsia (combined formulation) | Low evidence<br>Possibly reduces indigestion, bloating, spasms |
| *Psyllium* | May help maintain normal digestive system function and facilitate and improve digestion | Habitual constipation<br>LSTU | Contributes to intestinal transit and intestinal function<br>Helps to maintain a healthy bowel and facilitate intestinal transit<br>Digestion<br>Exact wording of claim as it appears on product: | Trials [72]: reduced constipation, bloating<br>In vivo on rats [73]: anti-ulcer<br>[74]; in overweight and obese individuals, reduced constipation and cholesterol levels | Low evidence regarding Gastric acid induced problems<br>Good evidence for constipation, source of fiber |

Table S1: Comparison of the identified health claims and their supporting evidence

| | | | | | |
|---|---|---|---|---|---|
| | | | psyllium seed for healthy digestion and healthy colon<br><br>Examples of any alternative wording that may be used in relation to claim: contributes to intestinal transit and intestinal function; contributes to stool softening and bowel regularity; it helps to control blood levels of cholesterol<br>“On hold” | | |
| *Caraway* | Supports digestive system function, stimulates digestion, reduces gas accumulation in the GI tract, and alleviates the feeling of heaviness in the abdomen | Reduction in gastrointestinal discomforts such as bloating and flatulence<br>LSTU | Supports digestion and digestive functions<br>Stimulates digestion<br>Relieves fullness and windy feelings<br>Contributes to the normal function of intestinal tract<br>Helps support the digestive juice flow<br>Helps with belly spasm<br>Helps to support digestion<br>“On hold” | Traditional use reports [21] an effect on increased stomach acid, burning sensations in the epigastrium, and various stomach and digestive disorders<br>Survey [79]: various stomach and digestive disorders<br>On humans [80]: reduction in gastrointestinal discomfort, dyspeptic symptoms, spasms<br>In vitro [80]: antibacterial effect on *H. pylori*<br>Trial [85]: pain relief in digestive system<br>Trial [85]: (combined preparation) reduced stomach pain | Low evidence<br>Possible effect reduction in gastric acid, pain relief, spasms, GI discomfort and dyspeptic symptoms, protects mucosa, antibacterial |
| *Caraway essential oil* | | | | In vitro [81]: antibacterial | |

Table S1: Comparison of the identified health claims and their supporting evidence

| | | | | | |
|---|---|---|---|---|---|
| | | | | Animal models [82]: antisecretory activity by reducing gastric acid secretion and maintaining physiological pH levels, protects gastric mucosa<br>In vitro [83]: reduced gastric acid and spasms<br>In vitro [84]: antioxidant | |
| *Liquorice* | Helps maintain normal digestive system function, supports the digestive process and immune system function, antioxidant | Relief of burning sensation and dyspepsia<br>LSTU | Active substances of licorice help maintain normal function of mucous membranes in the stomach and small intestine<br>Traditionally used to facilitate the digestion<br>Used to facilitate the digestion<br>Contributes to the digestive comfort<br>Helps to support the digestion<br>Contributes to support the digestion<br>Helps to maintain balance and comfort in the digestive systems of people with sensitive digestions<br>"On hold" | Clinical trials [88]: no significant effects in GI problem reduction<br>Trial [88] and in vitro [89]: antibacterial effects against *H. pylori*<br>In vivo mouse models of acute alcoholic gastric ulcer [91]: reduced ulcers | Low evidence<br>Possible future direction antibacterial against *H. pylori*, anti-ulcer |
| *Chicory* | Helps to maintain normal digestive function, supporting digestion, and promotes the | The relief of symptoms related to mild digestive disorders, such as a feeling of abdominal fullness, flatulence, and slow digestion, as well | Supports digestion<br>Contributes to the stimulation of the production of the digestif body fluids and of the gastro-intestinal movement<br>"On hold" | In vitro gastric model [96]: antioxidant, anti-inflammatory, high content of fiber | Low evidence<br>Mostly based on traditional use, associated with high content of fiber, antioxidant, anti- |

Table S1: Comparison of the identified health claims and their supporting evidence

| | | | | | |
|---|---|---|---|---|---|
| | release of digestive juices | as for temporary loss of appetite<br>LSTU | | | inflammatory properties |
| *Lemon balm* | Promotes digestion, supports regular GI motility, helps reduce gas that leads to bloating, has a positive impact in cases of indigestion and feelings of heaviness | Relief of mild symptoms of mental stress and to aid sleep, as well as for the symptomatic treatment of mild gastrointestinal complaints, including bloating and flatulence<br>LSTU | Supports digestion and digestive functions<br>Stimulates digestion<br>Relieves fullness and windy feelings<br>Contributes to the normal function of intestinal tract<br>Helps support the digestive juice flow<br>Helps with belly spasm<br>Helps to support digestion<br>"On hold" | In vivo [103]: reduction in gastric ulcers in mice<br>Ex vivo [102]: influence motility in the ileum and jejunum<br>Trial [104]: colic symptoms in infants<br><br>In vitro models [99]: anti-inflammatory, a strong ability to induce (antrum) contractility, improved motility<br>Traditional use [101]: digestive aid, fever reduction, flatulence, flatulent colic, GI disorders<br>In vitro [101]: antioxidant anti-inflammatory, antispasmodic<br>Trial [101]: colitis, dyspepsia (issue- combined preparations) | Low evidence<br>Gastric ulcers, motility, colic, spasms, dyspepsia |

**Abbreviations:**

LSTU—Long-Standing Use or Traditional Use

FS—Food Supplement

GI—Gastrointestinal

EMA—European Medicines Agency

EFSA—European Food Safety Authority

ICU—Intensive Care Unit
